# Effects of Maternal Psychopathology and Education Level on Neurocognitive Development in Infants of Adolescent Mothers Living in Poverty in Brazil

**DOI:** 10.1016/j.bpsc.2019.05.009

**Published:** 2019-10

**Authors:** Elizabeth Shephard, Daniel Fatori, Larissa Rezende Mauro, Mauro V. de Medeiros Filho, Marcelo Q. Hoexter, Anna M. Chiesa, Lislaine A. Fracolli, Helena Brentani, Alexandre A. Ferraro, Charles A. Nelson, Euripedes C. Miguel, Guilherme V. Polanczyk

**Affiliations:** a Department of Psychiatry, Faculdade de Medicina, Universidade de São Paulo, São Paulo, Brazil; b School of Nursing, Universidade de São Paulo, São Paulo, Brazil; c Institute of Psychiatry, Psychology, and Neuroscience, King’s College London, London, United Kingdom; d Laboratories of Cognitive Neuroscience, Division of Developmental Medicine, Boston Children’s Hospital, Harvard Medical School, Boston, Massachusetts; e Graduate School of Education, Harvard University, Cambridge, Massachusetts

**Keywords:** Adolescent motherhood, EEG, Infancy, Maternal psychopathology, Neurocognitive development, Oscillatory activity

## Abstract

**Background:**

Adolescent motherhood remains common in developing countries and is associated with risk factors that adversely impact infant neurodevelopment, including poverty, low maternal education, and increased maternal psychopathology. Yet, no published work has assessed how these factors affect early brain development in developing countries.

**Methods:**

This pilot study examined effects of maternal psychopathology and education on early neurocognitive development in a sample of adolescent mothers (*N* = 50, final *n* = 31) and their infants living in poverty in São Paulo, Brazil. Maternal symptoms of anxiety, depression, and attention-deficit/hyperactivity disorder and education level were assessed during pregnancy. Infant neurocognitive development was assessed at 6 months of age, with oscillatory power and functional connectivity in the theta (4–6 Hz), alpha (6–9 Hz), and gamma (30–50 Hz) frequencies derived from resting-state electroencephalography; temperament (negative affect, attention, and regulation); and cognitive, language, and motor skills. Cluster-based permutation testing and graph-theoretical methods were used to identify alterations in oscillatory power and connectivity that were associated with maternal psychopathology and education. Correlations between power and connectivity alterations were examined in relation to infants’ overt cognitive behavioral abilities.

**Results:**

Increased maternal anxiety and lower maternal education were associated with weaker oscillatory connectivity in alpha-range networks. Infants with the weakest connectivity in the alpha network associated with maternal anxiety also showed the lowest cognitive ability. Greater maternal anxiety and attention-deficit/hyperactivity disorder were associated with increased absolute and relative theta power.

**Conclusions:**

Our findings highlight the importance of addressing maternal psychopathology and improving education in poor adolescent mothers to prevent negative effects on infant neurodevelopment.

Adolescent motherhood remains common in developing countries and is associated with increased prevalence of risk factors that are known to adversely impact infant neurodevelopment. In Brazil, for example, the adolescent birth rate is 60.5 of 1000 compared with 14.4 of 1000 and 22.3 of 1000 in the United Kingdom and United States, respectively (1). These young mothers frequently live in impoverished environments and show high rates of mental health problems, including depression, anxiety, impulsivity, and suicidal behavior, which are often undetected and untreated by health care professionals 2, 3. These young women are often disconnected from school and find it increasingly difficult to continue their scholarship during pregnancy, resulting in low education levels 4, 5. Increased rates of neurodevelopmental disorders such as attention-deficit/hyperactivity disorder (ADHD) are found in adolescent mothers in developed countries (6), though this association has not been studied in developing countries. Outside of the context of adolescent motherhood, these factors have been robustly associated with poorer cognitive, language, and socioemotional development and increased rates of internalizing and externalizing disorders in offspring 7, 8.

Given the high rates of these risk factors among adolescent mothers in low-resource countries, infants born to these young mothers represent a high-risk population for impaired neurocognitive development. Yet, few studies have investigated the development of these infants. In Brazil, the handful of studies have reported poorer motor development in the first 18 months of life (9) and increased internalizing and externalizing behavior problems in toddlerhood (10) in infants of adolescent mothers. It is unclear, however, which specific risk factors contribute to these early developmental problems. Furthermore, the mechanisms by which these risks result in impaired infant development have not been investigated. One explanation for the latter question is that maternal psychopathology coupled with poverty and poor education alters early neurodevelopmental processes, particularly the formation of brain networks that provide the structure for acquiring motor, cognitive, and socioemotional skills (11). In turn, early disruptions to brain development lead to atypical developmental trajectories (12). Consistent with this account, atypicalities in brain structure, function, and functional connectivity in the first year of life have been associated with low socioeconomic background, maternal psychopathology, and low parental education in infants of adult mothers 13, 14, 15, 16.

To our knowledge, no published work has examined the effects of maternal risk factors on neurodevelopment in infants of adolescent mothers in developing countries. Understanding precisely how brain function is affected in these infants and which maternal risk factors brain alterations relate to, such as psychopathology or low education, will be crucial in designing targeted, effective interventions to prevent impaired development in these infants. This knowledge will also be important for supporting public policies designed to reduce social disadvantages from the beginning of life.

In this pilot study, we took the first steps to investigating these issues by examining how maternal psychopathology (symptoms of depression, anxiety, and ADHD) and education level affect early brain development in infants of adolescent mothers living in impoverished urban regions of São Paulo, Brazil. Infant brain function was assessed using resting-state electroencephalography (EEG) from which we derived measures of oscillatory power and phase synchronization (functional connectivity). In EEG, oscillatory power indexes the magnitude of activity across populations of neurons and is recorded at the scalp. The frequency characteristics of oscillatory power have been robustly linked with different neurocognitive processes in infants, older children, and adults 17, 18. For example, power in the mid-range alpha frequency (primarily 6–9 Hz in infants; 8–12 Hz in older individuals) is associated with cortical inhibition and attention 19, 20, 21, 22, low-frequency theta power (4–6 Hz in infants; 4–8 Hz in older individuals) is implicated in attentional and regulatory processes 22, 23, 24, 25, and high-frequency gamma power (>30 Hz) is associated with integrative perceptual processes and cognitive and language development 26, 27, 28. In infants, atypicalities in oscillatory power, including reduced alpha and gamma power and increased theta power, have been found in relation to maternal psychopathology, socioeconomic and psychosocial deprivation, and later-emerging developmental problems 13, 14, 29. Another important characteristic of oscillatory activity is phase synchronization, which reflects the extent to which signals from different neural populations oscillate in synchrony (with the same timing). Phase synchrony is believed to be a key neuronal communication mechanism that coordinates activity across different brain regions (functional connectivity), facilitating the formation of functional neural networks (30). While the specific brain regions involved in such networks cannot be localized with EEG owing to the technique’s low spatial resolution, the frequency at which phase synchronization occurs is, like oscillatory power, associated with particular neurocognitive functions 17, 31. Furthermore, disruptions to oscillatory networks, such as reduced phase synchrony in the alpha and gamma frequencies and atypically increased phase synchrony in the theta frequency, have been found to index exposure to adverse environments, maternal psychopathology, and early developmental problems in infancy 32, 33, 34. Oscillatory power and phase synchronization are therefore valuable methods for investigating the influence of maternal variables on neural activity and the temporal dynamics of neural networks in early neurodevelopment. Following previous work 13, 14, 21, 22, 23, 27, 28, 29, 31, 32, 33, 34, we focused on oscillatory power and phase synchronization (hereafter, “connectivity”) in the infant theta (4–6 Hz), alpha (6–9 Hz), and gamma (30–50 Hz) frequencies.

We used cluster-based permutation testing and graph theoretical methods to test the following hypotheses: 1) higher maternal psychopathology and lower maternal education would be associated with lower oscillatory power and connectivity in the alpha and gamma frequencies and with higher power and connectivity in the theta frequency in infants; 2) these alpha and gamma reductions and theta increases would in turn be associated with poorer cognitive, language, emotional, motor, and attentional and regulatory abilities in infants. Given the dearth of information on neurodevelopment in infants of adolescent mothers in developing countries, we also explored oscillatory activity patterns directly associated with infants’ developmental abilities. Based on previous research with infants of adult mothers, we expected stronger power and connectivity in the alpha and gamma frequencies to be associated with better cognitive, language, and motor abilities; for the theta frequency, we expected stronger power and connectivity (at different scalp regions to those associated with maternal psychopathology and education) to be associated with better attentional and regulatory abilities 21, 22, 27, 28.

## Methods and Materials

### Participants

Fifty adolescent mothers and their infants participated in this study. Adolescents were recruited during their pregnancies from primary health care units in poor western regions of São Paulo, Brazil. These regions are characterized by high rates of urban violence, widespread slums, and adverse living conditions (lack of sanitation, poverty). At recruitment, all adolescents were 14 to 19 years of age, pregnant for the first time, between 8 and 16 weeks’ gestation, living in western regions of São Paulo, and of low socioeconomic status (SES) [classes C–D/E according to the Criteria for Economic Classification assessed by the Brazilian Association of Research Companies questionnaire (35)]. The adolescents were taking part in a larger randomized controlled trial (NCT02807818) of a home visitation intervention designed to improve parenting skills and infant development (36). Twenty-five adolescents received the intervention and 25 received usual care. The current dimensional analyses were conducted on the intervention and control groups combined with intervention group covaried in analyses (see the Supplement for further details concerning effects of the intervention).

Infants completed EEG and cognitive behavioral assessments at 6 months of age. Two infants were born 1 month prematurely (retained in analysis); the remaining 48 infants were full term. All infants were without neurological or genetic problems. Usable EEG data were not acquired from all 50 infants (detailed below), and final analyses were conducted on a sample of *n* = 31. The infants who did and did not provide usable EEG data did not differ significantly in infant or maternal characteristics (see the Supplement for retention analysis). Characteristics of the full and analysis samples are shown in Table 1. Written informed consent was obtained from all adolescent mothers and, if <18 years of age, their parent or guardian. The study was approved by the ethical review boards of the University of São Paulo Medical School (ref: 052/15) and the São Paulo Municipal Health Department.

**Table 1:** Sample Characteristics

	Recruited Sample (*N* = 50)	Analysis Sample (*n* = 31)
Intervention/Control	25/25	17/14
Maternal Measures at Baseline		
Age, years	16.86 (1.37)	16.61 (1.54)
SES C/DE	32/18	19/12
Anxiety, BAI	10.68 (7.56)	12.29 (8.02)
Depression, BDI-II	13.22 (7.73)	14.45 (8.18)
ADHD, ASRS	24.30 (10.85)	25.74 (11.74)
Maternal education level: L1, L2, L3, L4, L5	0, 7, 38, 5, 0	0, 6, 22, 3, 0
Infant Measures at 6 Months		
Sex, girls/boys	22/28	15/16
Age, weeks	26.76 (1.12)	26.74 (1.15)
Bayley Cognitive Composite	101.70 (11.19)	100.48 (10.52)
Bayley Language Composite	88.76 (12.55)	90.42 (7.75)
Bayley Motor Composite	101.02 (11.50)	100.10 (11.34)
IBQ-R Negative Affect	3.96 (0.87)	4.03 (0.85)
IBQ-R Orienting/Regulation	5.13 (0.67)	5.08 (0.67)
EEG resting-state epochs	–	46.81 (18.93)

Values are presented as *n* or mean (SD).

ADHD, attention-deficit/hyperactivity disorder; ASRS, Adult ADHD Self-Report Scale total score; BAI, Beck Anxiety Inventory total score; Bayley, Bayley Infant Scales of Development III; BDI-II, Beck Depression Inventory II total score; EEG, electroencephalography; IBQ-R, Infant Behavior Questionnaire—Revised; L1, illiterate or elementary school incomplete, L2, elementary school complete or middle school incomplete; L3, middle school complete or high school incomplete; L4, high school complete or university incomplete; L5, university complete; SES C/DE, socioeconomic status class C or DE.

### Measures

#### Maternal Psychopathology and Education

Maternal psychopathology and education level were assessed at recruitment into the study. Education level was classified into 5 categories (see Table 1). Symptoms of anxiety and depression were assessed using the Brazilian Portuguese versions of the Beck Anxiety and Depression Inventories (37), which are 21-item rating scales that assess the presence and severity of anxiety and depression symptoms. Scores range from 0 to 63; higher scores reflect more severe symptoms. Maternal ADHD symptoms were assessed with the Brazilian Portuguese version of the Adult ADHD Rating Scale (38), which contains 18 questions measuring the frequency of recent ADHD symptoms and is based on DSM-IV-TR (39) diagnostic criteria for adult ADHD. Scores range from 0 to 72; higher scores indicate more severe ADHD. The Beck Anxiety and Depression Inventories and Adult ADHD Rating Scale were administered to adolescents in interview format by trained psychologists. The total score for each measure was used in analysis.

#### Cognitive Behavioral Measures of Infant Development at 6 Months

The Bayley Scales of Infant Development-Third Edition (40), a standardized instrument for infants 1 to 42 months of age, was administered to all infants by trained psychologists. Age-normed composite scores (mean [SD]: 100 [15]) for cognitive, language, and motor ability were used in analysis. Infant temperament was assessed with the Brazilian Portuguese version of the Infant Behavior Questionnaire–Revised (41) administered to mothers in interview format by psychologists. The Infant Behavior Questionnaire–Revised is a 191-item scale in which parents rate how often (never to always) their infant exhibits particular behaviors in everyday contexts. Items are averaged to yield subscales and factors indexing different temperament dimensions. In this study, the factors negative affect and orienting/regulation were used. Higher negative affect scores reflect greater negative emotions (fear, sadness, distress). Higher orienting/regulation scores reflect better attentional and regulatory abilities (longer attending or orienting to objects, higher soothability).

### Resting-State EEG at 6 Months

#### EEG Recording and Preprocessing

Infants completed a 3-minute resting-state EEG recording while sitting on their mother’s lap approximately 65 cm in front of a computer screen in a dimly lit room. A video of abstract shapes was shown throughout recording to engage infants’ attention. EEG data were recorded using a 128-channel Geodesic Sensor Net and a NetAmp 200 DC-coupled amplifier (Electrical Geodesics Inc., Eugene, OR). The data were referenced online to electrode Cz, sampled at 500 Hz, and bandpass filtered between 0.1 and 100 Hz. EEG data were preprocessed offline using Brain Vision Analyzer version 2.1 (Brain Products, Munich, Germany). Electrodes around the rim of the net were contaminated by excessive artifacts and removed from all participants, leaving 80 electrodes in the analysis. The data were filtered using 0.1-Hz high-pass, 60-Hz low-pass 24-dB/oct Butterworth filters with a 60-Hz notch filter for residual electrical line noise. Periods of data with excessive noise or during which the infant was crying or reacting to external stimuli were excluded. Remaining flat or noisy channels were removed and interpolated using spherical spline interpolation prior to re-referencing to the average reference. Independent components analysis was used to identify and remove ocular artifact components, after which the data were segmented into 2-second nonoverlapping epochs. Epochs with remaining artifacts (amplitudes ± 150 μv) were excluded. Infants with fewer than 20 artifact-free epochs were excluded from analysis. Lastly, a Laplacian transform using spherical splines (42) (lambda: 1 × 10^−5^; order of splines: 4; degree of Legendre polynomials: 20) was applied to control for volume conduction.

EEG data from 14 infants were unusable owing to technical problems with the EEG system that resulted in corrupted data files (*n* = 4) or poor-quality recordings (*n* = 10). A further 5 infants had fewer than 20 artifact-free epochs for analysis and were also excluded, leaving a final sample for analysis of *n* = 31 mother-infant dyads (17 in the intervention group and 14 in the control group).

#### Computation of Oscillatory Power and Connectivity

The cleaned, Laplacian-transformed epochs were exported to FieldTrip (43) within the MATLAB R2017B (The MathWorks, Inc., Natick, MA) environment for computation and analysis of oscillatory power and connectivity. In FieldTrip, the 2-second epochs were subjected to fast Fourier transform with a 10% Hanning window taper to obtain power estimates and Fourier coefficients for the 1- to 50-Hz range at 1-Hz intervals. Power estimates were averaged across frequency steps in the theta (4–6 Hz), alpha (6–9 Hz), and gamma (30–50 Hz) ranges (see the Supplement for further information on frequency bands), resulting in power spectra containing the average absolute theta, alpha, and gamma power at each electrode. Relative power spectra were also computed (absolute theta/alpha/gamma band power divided by absolute 1- to 50-Hz broadband power). Absolute and relative power spectra (6 per participant) were used in statistical analyses.

Oscillatory connectivity (phase synchronization) was quantified by the debiased weighted phase lag index (dwPLI) [see (44) and the Supplement], computed from Fourier coefficients at each 1- to 50-Hz frequency step between each pair of electrodes across epochs. This resulted in one 80 × 80 adjacency matrix per frequency step per participant, where matrix element *ij* holds the phase synchronization (dwPLI) of signals between *i* and *j* electrodes. Adjacency matrices were averaged across frequency steps to obtain one 80 × 80 adjacency matrix for connectivity in the theta, alpha, and gamma frequency bands. The resulting matrices (3 per participant) were used in statistical analysis.

### Statistical Analysis

The hypothesis that maternal psychopathology and education would be associated with decreases (alpha/gamma) and increases (theta) in infants’ oscillatory activity (hypothesis 1) was tested by examining associations between maternal variables (anxiety, depression, ADHD, education level) and infants’ oscillatory power (relative and absolute theta, alpha, and gamma power) and connectivity (dwPLI in theta, alpha, and gamma bands). For absolute and relative power, cluster-based permutation testing (45) was conducted in FieldTrip to identify clusters of electrodes at which power was significantly (*p* < .05) positively or negatively associated with maternal psychopathology and education while controlling for multiple comparisons (80 power values per power spectra). For oscillatory connectivity, the network-based statistic (NBS) (46) was used to identify oscillatory neural networks, defined according to graph theory (47) as topologically connected clusters of nodes (electrodes) based on the strength of their edges (oscillatory synchrony between electrodes, dwPLI), that were significantly (*p* < .05) associated with maternal psychopathology and education while controlling for multiple comparisons (6400 dwPLI values per adjacency matrix). Significant brain networks were visualized with BrainNet Viewer (48). Further details of the cluster-based permutation and NBS methods are provided in the Supplement. Additionally, whole-brain connectivity, defined as the average dwPLI across all electrodes, was computed for each frequency band (theta, alpha, gamma) and used as an outcome variable in regression models, with maternal variables predicting whole-brain connectivity (all variables were mean-centered prior to regression analysis). All analyses were conducted while covarying group (intervention, control) to control for possible intervention effects. Subsequently, analyses were repeated covarying maternal age (years) to control for biological maturity of the mother, infant age (weeks, corrected for prematurity) to control for age-related changes in neural activity, and SES to control for variability in socioeconomic deprivation. We also modeled interactions with group (see the Supplement for details).

The hypothesis that greater decreases (alpha/gamma) and increases (theta) in oscillatory power and connectivity would be associated with poorer cognitive behavioral development (hypothesis 2) was tested by first computing the average power in clusters and average connectivity in networks that were significantly associated with maternal variables. Next, partial Spearman correlations were computed between those power and connectivity metrics and infant cognitive ability, language ability, motor ability, attention and regulatory ability, and negative affect. Correlations were computed controlling for group and repeated controlling for maternal and infant age and SES.

Our exploratory analysis examining oscillatory activity directly associated with infants’ cognitive-behavioral development was conducted using cluster-based permutation testing, NBS, and regression (as described above for hypothesis 1) to identify patterns of power and connectivity that were related to infants’ cognitive, language, motor, and attention and regulatory ability and their level of negative affect.

## Results

### Infant Oscillatory Power and Maternal Psychopathology and Education

**Figure 1:**
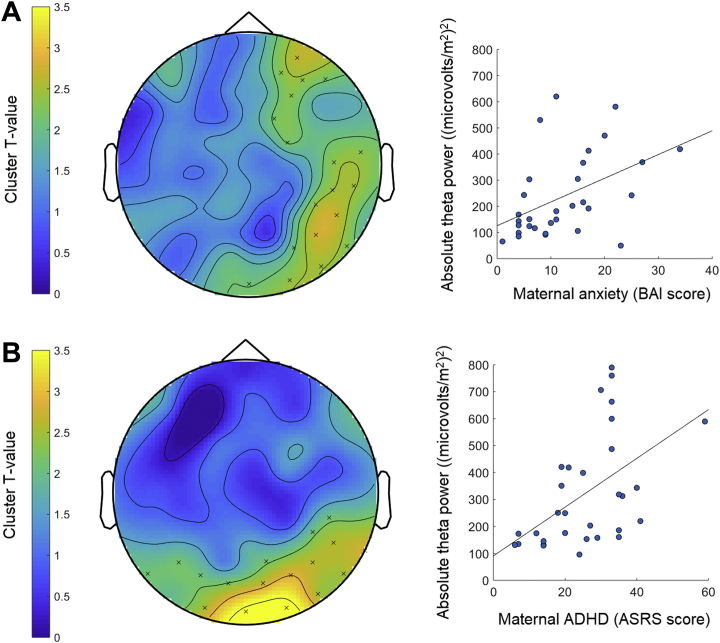
Associations between infant absolute power and maternal anxiety and attention-deficit/hyperactivity disorder (ADHD) symptoms. Topographical plots (left) show the clusters of electrodes (indicated by × signs) at which infants’ absolute oscillatory power in the theta frequency was significantly positively associated with maternal anxiety **(A)** and ADHD **(B)** symptoms. Color bars represent the statistical strength (*T* value) of the association between oscillatory power at different scalp regions and maternal anxiety or ADHD, with higher *T* values reflecting a stronger statistical association. Scatterplots (right) display the positive associations between absolute theta power in the significant clusters and maternal anxiety **(A)** and ADHD **(B)**. Note that absolute power values are large owing to the Laplacian transform applied to the data prior to computation of spectral power (μv^2^). ASRS, Adult ADHD Self-Report Scale; BAI, Beck Anxiety Inventory.

**Figure 2:**
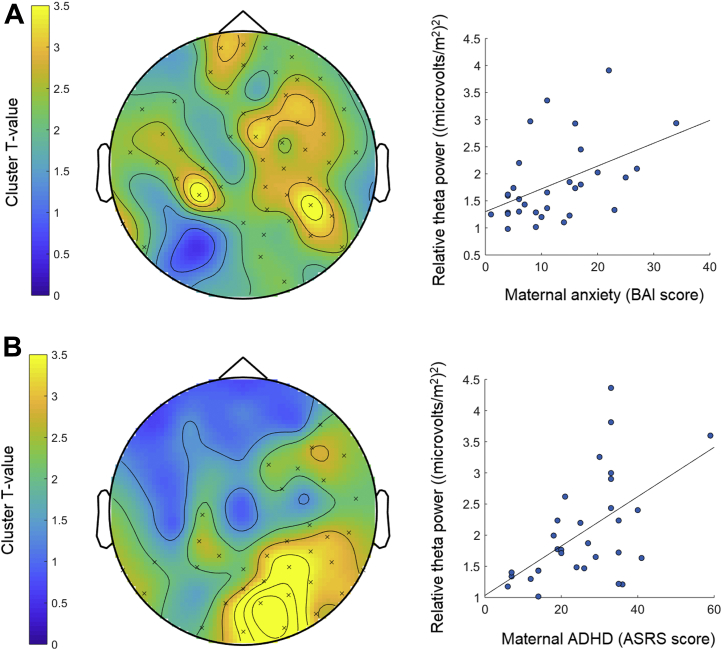
Associations between infant relative power and maternal anxiety and attention-deficit/hyperactivity disorder (ADHD) symptoms. Topographical plots (left) show the clusters of electrodes (indicated by × signs) at which infants’ relative oscillatory power in the theta frequency was significantly positively associated with maternal anxiety **(A)** and ADHD **(B)** symptoms. Color bars represent the statistical strength (*T* value) of the association between oscillatory power at different scalp regions and maternal anxiety or ADHD, with higher *T* values reflecting a stronger statistical association. Scatterplots (right) display the positive associations between absolute theta power in the significant clusters and maternal anxiety **(A)** and ADHD **(B)**. ASRS, Adult ADHD Self-Report Scale; BAI, Beck Anxiety Inventory.

Cluster-based permutation testing revealed significant positive associations between absolute theta power at frontal and posterior electrode clusters and maternal anxiety (*p* = .04) and absolute theta power at posterior clusters and maternal ADHD symptoms (*p* = .03) while controlling for group (Table 2, Figure 1). Similar positive associations were revealed between relative theta power at frontocentral clusters and maternal anxiety (*p* = .009) and relative theta power at frontal and posterior clusters and maternal ADHD symptoms (*p* = .008) while controlling for group (Table 2, Figure 2). These effects remained significant while controlling for maternal and infant age and SES (*p* < .05). There were no further significant associations and no interactions with group (*p* > .06).

**Table 2:** Statistical Test Results for Cluster-based Permutation Tests and Network-Based Statistic

Significant Association (Covarying Group)	*df*	Cluster Statistic (Effect Size)^a^	Network Statistic (Effect Size)^b^
Maternal Anxiety and Infant Absolute Theta Power	30	44.83 (0.42)	–
Maternal ADHD and Infant Absolute Theta Power	30	43.55 (0.46)	–
Maternal Anxiety and Infant Relative Theta Power	30	134.18 (0.46)	–
Maternal ADHD and Infant Relative Theta Power	30	102.57 (0.51)	–
Maternal Anxiety and Infant Alpha Connectivity	30	–	3.47 (0.62)
Maternal Education and Infant Alpha Connectivity	30	–	3.41 (0.61)
Infant Negative Affect and Relative Alpha Power	30	17.75 (0.40)	–

ADHD, attention-deficit/hyperactivity disorder.

Dashes indicate that cluster or network statistics were not reported because the association was not significant. See the Supplement for full details of the procedure for computing the cluster and network statistics and effect size estimates.

a The maximum cluster statistic (summed cluster statistics) that was subjected to permutation testing in cluster-based permutation tests and its effect size (Cohen’s *d* estimate).

b The average network statistic for the significant connections in networks identified by network-based statistic and its effect size (Cohen’s *d* estimate).

### Infant Oscillatory Connectivity and Maternal Psychopathology and Education

**Figure 3:**
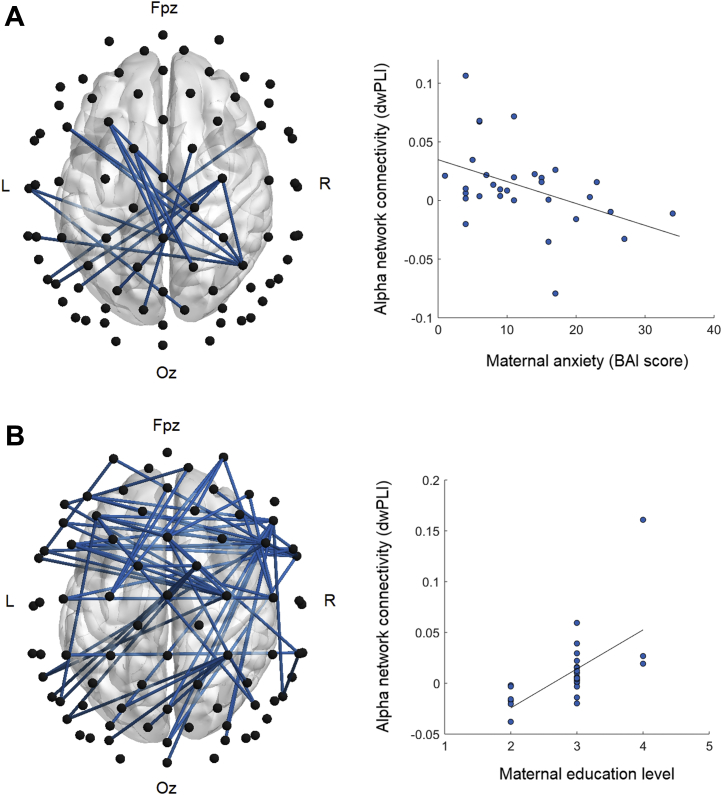
Associations between infant oscillatory network connectivity and maternal anxiety and education level. **(A)** Connectivity, quantified by debiased weighted phase lag index (dwPLI), was significantly negatively associated with maternal anxiety in the oscillatory network (left) in the alpha frequency. Black circles represent the nodes (electrodes) of the network and blue lines represent the edges (functional connections between nodes, indexed by oscillatory synchrony–dwPLI). All edges in the network showed reduced dwPLI (reduced connectivity) in association with greater maternal anxiety symptoms. The networks were visualized using BrainNet Viewer (48). The scatterplot (right) displays the negative association between connectivity (average dwPLI in the network) and maternal anxiety symptoms. Note that the dwPLI can take small negative values due to the debiasing procedure. **(B)** The oscillatory network in the alpha frequency (left) was significantly associated with maternal education level. All connections in this network showed weaker connectivity (lower dwPLI values) in association with lower levels of maternal education, as illustrated in the scatterplot to the right. BAI, Beck Anxiety Inventory; L, left; R, right.

NBS revealed networks in the alpha range in which connectivity was significantly associated with maternal anxiety and education level while controlling for group; weaker connectivity in these networks was associated with greater maternal anxiety (*p* = .03) and lower maternal education (*p* = .01) (Table 2, Figure 3). The network associated with anxiety remained significant when controlling for maternal age (*p* = .04), marginal when covarying SES (*p* = .055), but nonsignificant when covarying infant age (*p* = .13). The network associated with maternal education remained significant when covarying maternal and infant age and SES (*p* < .02). There were no further significant associations and no interactions with group (*p* > .06). Whole-brain connectivity was not associated with maternal variables (*F* < 2.49, *p* > .10).

### Infant EEG and Cognitive Behavioral Abilities

**Figure 4:**
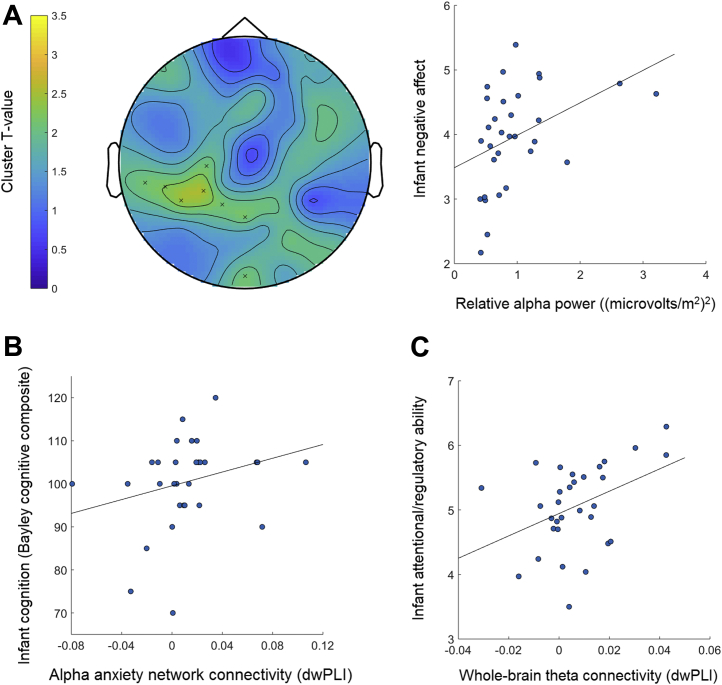
Associations between infant electroencephalography and cognitive-behavioral abilities. **(A)** The positive association between infants’ relative alpha power and level of negative affect is shown; the topographical plot displays the clusters of electrodes (indicated by × signs) at which this association was significant (color bar indicates the statistical strength—*T* value—of this association) and scatterplot displays the positive association between relative theta power in the significant clusters and infant negative affect scores. **(B)** Significant association between infant cognition and connectivity in the alpha-range network that was associated with maternal anxiety symptoms; weaker connectivity was associated with lower cognitive ability. **(C)** The plot shows the significant association between infant attentional and regulatory ability and whole-brain theta connectivity; better attention and regulation was associated with stronger connectivity across all electrodes. Note that the debiased weighted phase lag index (dwPLI) can take small negative values due to the debiasing procedure.

Partial Spearman correlations (controlling for group) were computed between power and connectivity measures that were significantly associated with maternal psychopathology and education (average absolute and relative theta power in clusters associated with maternal anxiety and ADHD, average connectivity in alpha-range networks associated with maternal anxiety and education level) and infants’ cognitive, language, motor, and attentional and regulatory abilities and negative affect. There was a significant association between infant cognitive ability and connectivity in the alpha-range network that was related to maternal anxiety (ρ_28_ = .377, *p* = .04); infants with weaker connectivity in this network had lower cognitive skills (Figure 4A). This association remained significant when controlling for maternal and infant age and SES (ρ > .462, *p* < .01). There were no further significant associations (ρ < .334, *p* > .07).

We next examined how oscillatory power and connectivity were associated with infants’ cognitive-behavioral abilities directly. Cluster-based permutation testing showed that increased negative affect was associated with greater relative alpha power at central clusters (*p* = .04 covarying group [see Table 2, Figure 4B]; *p* < .04 covarying infant and maternal age and SES). NBS revealed no significant networks associated with cognitive-behavioral abilities (all *p* > .06). However, whole-brain connectivity in the theta range was positively associated with infants’ attentional and regulatory ability (*F*_2,28_ = 3.42, *p* = .047, adjusted *R*^*2*^ = .15 covarying group), with stronger theta connectivity across all electrodes associated with better attention and regulation (β *=* .44, *t* = 2.60, *p* = .015) (Figure 4C); this association remained significant when covarying infant and maternal age (*F* > 2.93, *p* < .05) and at trend level when covarying SES (*F* = 2.60, *p* = .07). There were no further significant associations and no interactions with group (*p* > .08).

## Discussion

This pilot study examined effects of maternal psychopathology and education on neurodevelopment in infants of poor adolescent mothers living in poverty in Brazil. Increased maternal anxiety and lower maternal education were associated with weaker connectivity in oscillatory networks in the alpha frequency. Because connectivity in functional brain networks increases over the first year of life in typical development 31, 49, our findings of reduced connectivity in association with increased maternal anxiety and lower maternal education suggest that these maternal factors disrupt this process, perhaps hindering the formation of functional neural networks. Importantly, infants with the weakest connectivity in the network associated with maternal anxiety also showed the lowest cognitive ability. This finding suggests that the disrupted connectivity related to maternal anxiety had a negative impact on infants’ overt neurocognitive ability, although the direction of causality cannot be determined from the current analyses. These connectivity findings are in line with previous work reporting altered alpha activity in infants of anxious mothers (14) and reduced structural brain development in infants of parents with lower education (16). Our study is the first, however, to report that these maternal factors are associated with reduced functional connectivity in oscillatory brain networks in infants of poor adolescent mothers. That these disruptions were restricted to the alpha frequency is informative about the function of the underconnected networks. Alpha oscillations play a key role in cortical inhibition and are likely mediated by inhibitory gamma-aminobutyric acidergic signaling (19). Evidence indicates that inhibitory neuronal signaling is fundamental for cortical reorganization and guiding functional neural network formation early in neurodevelopment (50). The weaker alpha connectivity associated with maternal anxiety and low education may therefore reflect disruptions to inhibitory signaling mechanisms and the architectural roles they play in brain circuit development.

We also found that higher levels of maternal anxiety and ADHD symptoms were associated with increases in absolute and relative theta power in infants. These findings are consistent with previous reports of increased theta power in infants of adult anxious mothers (51) and, though no published work has examined effects of maternal ADHD on infant brain function, with reports of elevated theta power indexing genetic risk for ADHD in adolescents (52). Given the role of theta oscillations in attentional and regulatory processes 22, 23, 24, 25 and the involvement of these cognitive functions in anxiety and ADHD 53, 54, 55, the current findings may reflect a vulnerability to emotion and attention regulation problems that is shared between maternal risk for anxiety and ADHD. However, these patterns of theta power were not correlated with infants’ overt attentional and regulatory ability or levels of negative emotionality. Still, perhaps at this early point in infancy, alterations in oscillatory power associated with maternal ADHD and anxiety do not yet translate to alterations in overt behavior. It will be important to investigate how these early oscillatory atypicalities relate to longer-term developmental abilities.

Our exploratory analysis revealed patterns of oscillatory power and connectivity associated with infants’ developing cognitive-behavioral abilities that are consistent with findings in other populations of infants. Infants with higher attentional and regulatory abilities showed stronger whole-brain connectivity in the theta range, in line with findings in infants of adult mothers reporting increased theta oscillations during attentional control 22, 23. Infants with higher levels of negative affect showed increased relative alpha power, consistent with previous findings in infants of adult mothers (56). Our findings add to the infant development field by showing that oscillatory dynamics associated with early neurocognitive abilities are replicable in previously unstudied samples of infants from developing countries.

### Clinical Implications

Our findings provide the first empirical (albeit suggestive) evidence from developing countries indicating that pregnant adolescents’ mental health problems are associated with altered development of their infants’ oscillatory neural activity in the first 6 months of life. A recent epidemiological study in Southern Brazil reported that 22.5% of pregnant adolescents met diagnostic criteria for at least 1 mental disorder and that symptoms were undetected (and untreated) in 80% of cases (2). The current findings highlight the importance of addressing this situation so that these young mothers receive appropriate mental health interventions, not only to improve their own health and well-being but also to prevent adverse effects on their infants’ neurodevelopment. In addition to targeting maternal mental health problems, our findings indicate that interventions for young mothers in developing countries should focus on improving education. Clearly, depending on their age, adolescent mothers will not have had the opportunity to complete the higher levels of their education. Still, evidence suggests that Brazilian pregnant adolescents have poorer educational attainment than their age-matched peers 4, 5, and in our sample, the mean age of the mothers who had only completed elementary school was only slightly younger than those who had completed middle school (15.8 vs. 16.5 years). Our findings could also be used to support public policies designed to reduce inequality and the challenges adolescent mothers in developing countries face, including poverty and lack of social support, which may in turn reduce the likelihood that these girls experience mental health problems and improve their infants’ development.

### Limitations

Our modest sample size and high data loss precluded more complex statistical modeling. Consequently, we could not examine interactive relationships between maternal factors and infant neurodevelopment. Future work in larger samples is needed to replicate our findings and model different pathways between maternal and infant variables. Relatedly, our analysis of interactions with intervention group were likely underpowered. We did not examine how factors such as maternal sensitivity affected associations between maternal risk factors and infant neurodevelopment. This is important because previous work indicates that maternal sensitivity can mediate associations between risk factors and infant development in adolescent mothers (57). Our infant data were from one time point only, and future longitudinal work is needed to examine whether early disruptions to infant brain function associated with maternal risk factors lead to altered developmental trajectories.

### Conclusions

This pilot study provides the first evidence from developing countries that maternal psychopathology and low maternal education are associated with alterations in oscillatory neural activity in infants of adolescent mothers. These findings could be used to tailor appropriate interventions and to support public policies aimed at alleviating social disadvantages in vulnerable groups.
