# Supplementary material for: Effects of Maternal Psychopathology and Education Level on Neurocognitive Development in Infants of Adolescent Mothers Living in Poverty in Brazil
Source: Biol Psychiatry Cogn Neurosci Neuroimaging. 2019 Oct;4(10):925–34. doi: 10.1016/j.bpsc.2019.05.009 (PMC6863387; doi:10.1016/j.bpsc.2019.05.009)
Supplement: Supplemental Material [file mmc1.pdf]

Effects of Maternal Psychopathology and Education Level on Neurocognitive Development in Infants of Adolescent Mothers Living in Poverty in Brazil

Supplemental Information

Retention analysis

Of the 50 infants taking part in this study, 31 (62%) provided usable EEG data and were included in analysis. Possible retention biases were investigated by comparing maternal and infant characteristics between the infants who did (n = 31) and did not (n = 19) provide usable EEG data. Maternal characteristics included symptoms of depression and anxiety assessed with the *Beck Depression and Anxiety Inventories* (*BAI & BDI*; 1), ADHD symptoms measured by the *Adult ADHD Self-Report Scale* (*ASRS*; 2), maternal level of education (classified in 5 categories ranging from *L1 – did not complete elementary school* to *L5 – completed university*), socio-economic status (classified as C/D or E according to the *Criteria for Economic Classification*, assessed by the *Brazilian Association of Research Companies questionnaire*; 3) and age (years) at recruitment. Infant characteristics (at age 6 months) included cognitive, language and motor ability assessed by the Cognitive, Language and Motor Composite scores on the *Bayley Scales of Infant Development-Third Edition* (4), level of negative emotions and attentional/regulatory ability measured with the Negative Affect and Orienting/Regulation factor scores of the *Infant Behavior Questionnaire – Revised* (*IBQ-R*; 5), sex, and age (weeks) at the 6-month assessment. A MANOVA comparing continuous variables between the EEG and no-EEG groups revealed no significant differences in maternal psychopathology, maternal/infant age, or infant cognition and behaviour (all $F \leq 3.13$, all $p \geq .08$, all $\eta p^2 \leq .064$). Similarly, Chi-square tests comparing categorical variables between the EEG and no-EEG groups revealed no significant differences in maternal socio-economic status, education level, or infant sex (all $\chi^2 \leq .637$, all $p \geq .31$).

Frequency band selection

We selected the theta (4-6Hz), alpha (6-9Hz) and gamma (30-50Hz) frequency bands for oscillatory power and connectivity analysis because these bands have been most robustly linked with cognitive functions and with effects of maternal psychopathology, adverse early environments and developmental problems in infants (see main text Background). To confirm our selection of these frequency bands was appropriate for our sample, we evaluated whether oscillatory power showed the typical frequency characteristics of infants of this age (i.e. a peak in power in the lower alpha range) and whether connectivity was particularly strong in a frequency band that we did not include in analysis. As Figure S1 shows, grand averaged power across all participants showed the typical alpha peak at ~7Hz, supporting our use of the predefined frequency bands in our sample. Figure S2 shows that the strength of connectivity (averaged across all participants and electrodes) varied little across the 1-50Hz frequency range, although we did see a peak in connectivity between 46-48Hz. Thus, our analysis focusing on the theta, alpha and gamma bands either include the frequency range containing the strongest connectivity (gamma band) or do not differ appreciably from the strength of connectivity in bands we did not examine (theta and alpha vs delta and beta). Since it is not recommended to calculate and analyse the dwPLI measure across narrow frequency bands because it can be unreliable (see e.g. 6) we did not conduct a supplementary analysis of the narrow-band 46-48Hz connectivity.

Figure S1 Average absolute power across participants plotted by 1Hz frequency step at site Cz

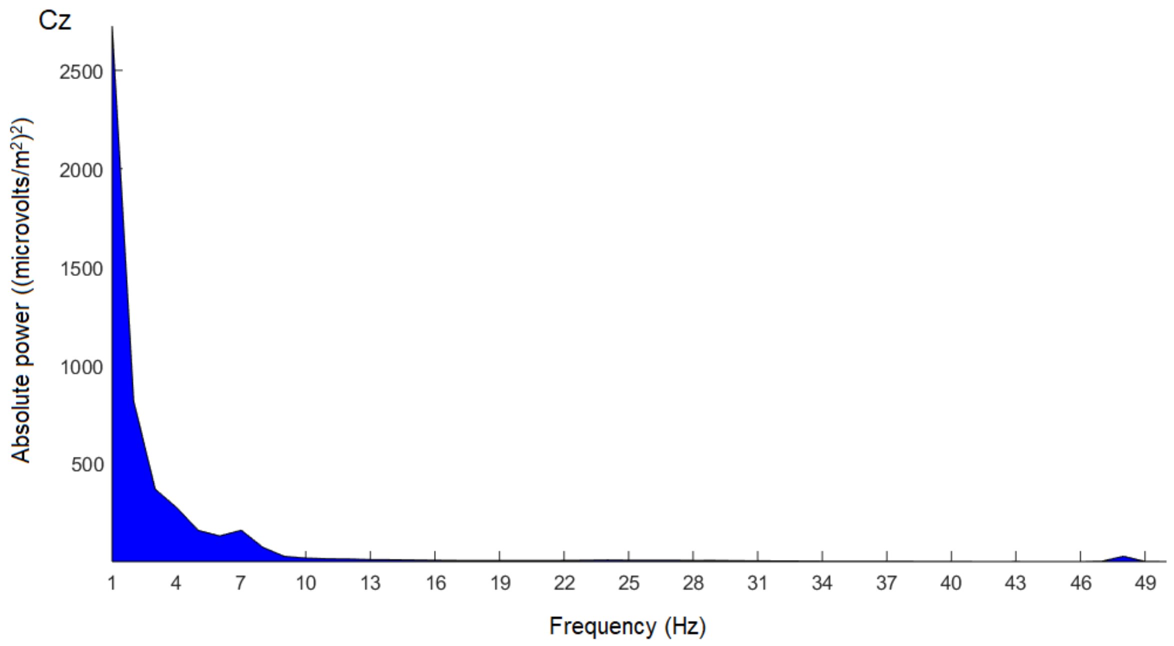

Figure S2 Average connectivity (dwPLI) across all electrodes and participants plotted by 1Hz frequency step

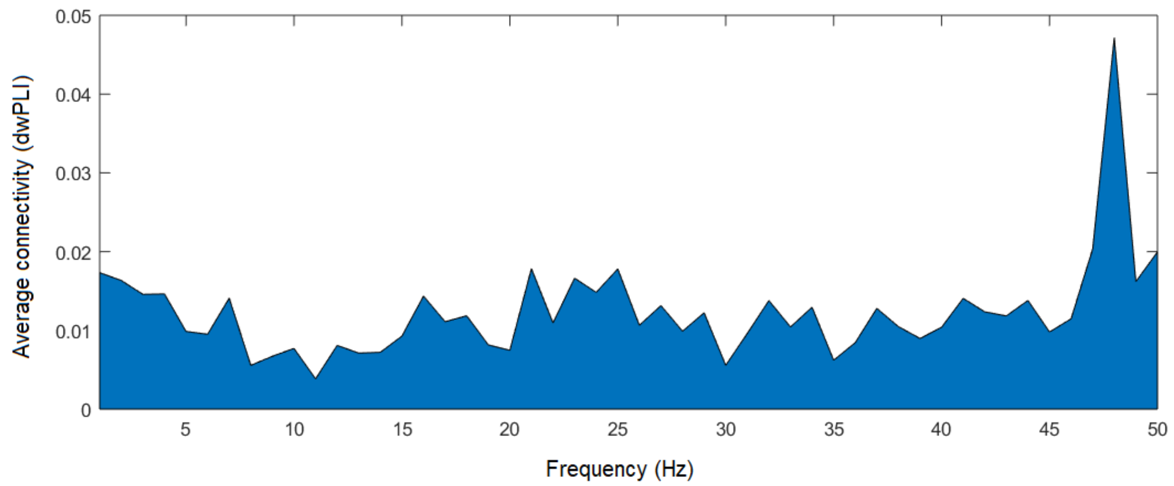

The debiased weighted phase lag index (dwPLI)

The debiased weighted phase lag index (dwPLI; 7) is an extension to a widely used measure of phase synchronisation, the phase lag index (PLI; 8). The PLI and dwPLI quantify phase synchronisation in terms of the extent to which two signals are coupled as reflected by the consistency of their (non-zero) phase lags (see (7,8) for further information). Both

measures range between 0 and 1, with values of 0 indicating the absence of phase synchronisation between the signals and values of 1 indicating perfect synchronisation between the signals. Extensions to the PLI introduced with the dwPLI include additional procedures to control for volume conduction and a debiasing procedure to correct for positive bias introduced by small sample sizes; as a consequence of the debiasing process, dwPLI values can sometimes take small negative values (see Figures 12-13 and description of the debiased weighting procedure on pages 1556-1557 of (7)). The negative-to-positive scale of the dwPLI can be interpreted in the same way as the traditional 0 to 1 scale, with smaller dwPLI values (e.g. those in the negative range) indicating weaker phase synchronisation and higher values (e.g. those above 0) reflecting stronger phase synchronisation.

Cluster-based permutation testing

Cluster-based permutation testing was used to identify clusters of electrodes at which oscillatory power in the theta, alpha and gamma frequencies was significantly positively or negatively associated with maternal psychopathology and education (first stage of analysis) and infant cognitive-behavioural measures (second stage of analysis). For each association of interest (e.g. theta power and maternal anxiety), the association between power at each electrode and the variable of interest was calculated to obtain a set of regression coefficients T. A threshold corresponding to $p < .05$ was applied to this set of coefficients to identify suprathreshold associations, which were then clustered into connected sets based on spatial adjacency. Cluster-level statistics from those sets were obtained by summing the T-values in each cluster and the resulting maximum cluster statistic was subjected to Monte-Carlo non-parametric permutation testing (using 1000 permutations) to obtain a p-value indicating significance level. This analysis method controls for multiple comparisons (i.e. the 80 power values from each of the 80 electrodes in power spectra) by reducing the data into spatially-

related clusters of electrodes, which are then subjected to statistical analysis (for further details see the tutorial paper presenting this method by (9)). In the main text, for each significant cluster-based permutation test, we report the maximum cluster statistic and its permutation p-value as well as an estimate of effect size; effect size (Cohen's d) was calculated by dividing the average cluster statistic by the square root of the sample, as suggested by Zalesky (personal communication, 2019).

Network based statistic (NBS)

NBS was used to identify oscillatory brain networks that were significantly positively or negatively associated with maternal psychopathology and education (hypothesis 1) and infant cognitive-behavioural variables (exploratory analysis). NBS first computes the association between each connection in adjacency matrices and the variable of interest resulting in a set of test statistics T, applies a primary threshold (T-value) to isolate connections with suprathreshold values, identifies topologically connected components (brain networks) among suprathreshold connections, and finally ascribes a p-value to identified networks via permutation testing. The choice of primary threshold T influences the likelihood of finding significant networks and the size (number of connections) in those networks. In the current study, we tested a range of primary thresholds: $T = 2.0$ (corresponding to $p \leq .05$), $T = 2.5$ (equivalent to $p < .018$), $T = 3.0$ (equivalent to $p < .005$), $T = 3.5$ (equivalent to $p < .002$) and $T = 3.6$ (corresponding to $p < .001$). Only networks that were significant at all primary thresholds were considered reliable and are reported in the main text Results; for those 'reliable' networks, we report the network connections and p-values for the primary threshold of $T = 3.0$ ($p < .005$) since this threshold yielded networks with neither extensive nor very few connections while providing strong statistical control. Preliminary analysis at the lowest primary threshold revealed dense, over-connected brain networks ($\geq$ 78 nodes, $\geq$ 120 edges)

associated with variables of interest which may have reflected spurious connectivity; thus, an additional thresholding step was conducted in which all connections with non-significant (i.e. not significantly different from zero) connectivity values were excluded (set to zero) from adjacency matrices prior to NBS, as suggested in Zalesky et al. (10). In the main text, for each significant network identified with NBS, we report the average network statistic (T-value) for the significant connections in each network, the permutation p-value for the network as well as an estimate of effect size; effect size (Cohen's d) was calculated by dividing the average network statistic by the square root of the sample, as suggested by Zalesky (personal communication, 2019).

Intervention effects

All adolescent mothers in this study were taking part in a randomised controlled trial (RCT, trial number NCT0280718) of a nurse home visitation intervention designed to improve mothers' parenting skills and address their health needs and improve infant development (11). Of the 50 adolescent mothers included in the current analyses, 25 received the intervention and 25 care-as-usual. A range of assessments of maternal mental health and infant development were conducted at regular intervals (recruitment and 3, 6, 12, 18 and 24 months post-partum). Analyses examining the effects of the intervention on both maternal mental health and infant neurocognitive development over this two-year period are currently in preparation for publication (Fatori et al., *In Preparation*). In the current study, our focus was on understanding how baseline levels of maternal psychopathology and education level affect infants' oscillatory brain dynamics in the first months of life, an issue that has not been studied previously in infants of adolescent mothers in developing countries. For these reasons, we have not included effects of the intervention on infants' oscillatory power and connectivity in the main analyses

of our current paper. However, since readers may be interested to know whether the intervention influenced infants' oscillatory activity, we summarise these analyses here.

Effects of the intervention were investigated by comparing oscillatory power (relative and absolute) and connectivity (connectivity in networks and whole-brain connectivity) in the theta, alpha and gamma bands between the Intervention (n = 17) and Control (n = 14) groups. Cluster-based permutation testing was used to compare oscillatory power between groups; network based statistic (NBS) was used to assess the presence of networks in which connectivity differed between groups. For oscillatory power, there were no clusters in which absolute or relative power differed significantly between groups (all $p \geq .99$). Likewise, for oscillatory connectivity, there were no networks in which connectivity differed significantly between groups (all $p \geq .20$).

While there were no effects of the intervention on oscillatory activity, it is possible that the intervention may have affected associations between maternal psychopathology and education and infants' brain development. Therefore, all analyses reported in the main text were conducted while covarying Group. In addition, we examined whether intervention Group interacted with maternal variables in associations with EEG activity. Specifically, we computed interaction terms between each maternal variable and Group (e.g. maternal anxiety x Group) and assessed whether this interaction term was significantly associated with infants' oscillatory power and connectivity. As reported in the main text, associations between maternal psychopathology and education and infants' power and connectivity were significant while controlling for Group and none of the interactions with Group were significant.

Supplemental References

1. Beck, A.T. & Cunha, J.A. (2001). Manual da versão em português das Escalas Beck. *São Paulo, Brazil: Casa do Psicólogo, 256.*
2. Kessler, R.C., Adler, L., Ames, M., Demler, O., Faraone, S., Hiripi, E.V.A., ... & Ustun, T.B. (2005). The World Health Organization Adult ADHD Self-Report Scale (ASRS): a short screening scale for use in the general population. *Psychological medicine*, *35*(2), 245-256.
3. Associação Brasileira de Empresas de Pesquisa (ABEP) (2007). Critério de classificação econômica Brasil. *Retrieved from:* http://www.abep.org/Servicos/Download.aspx?id=07
4. Bayley, N. (2006). *Bayley scales of infant and toddler development*. San Antonio, Texas: The Psychological Incorporation.
5. Gartstein, M.A., & Rothbart, M.K. (2003). Studying infant temperament via the revised infant behavior questionnaire. *Infant Behavior and Development*, *26*(1), 64-86.
6. Cohen, M.X. (2015). Effects of time lag and frequency matching on phase-based connectivity. *Journal of Neuroscience Methods*, *250*, 137-146.
7. Vinck, M., Oostenveld, R., Van Wingerden, M., Battaglia, F., & Pennartz, C.M. (2011). An improved index of phase-synchronization for electrophysiological data in the presence of volume-conduction, noise and sample-size bias. *Neuroimage*, *55*(4), 1548-1565.
8. Stam, C.J., Nolte, G., & Daffertshofer, A. (2007). Phase lag index: assessment of functional connectivity from multi channel EEG and MEG with diminished bias from common sources. *Human brain mapping*, *28*(11), 1178-1193.
9. Maris, E., & Oostenveld, R. (2007). Nonparametric statistical testing of EEG-and MEG-data. *Journal of neuroscience methods*, *164*(1), 177-190.

10. Zalesky, A., Fornito, A., & Bullmore, E.T. (2010). Network-based statistic: identifying differences in brain networks. *Neuroimage*, *53*(4), 1197-1207.

11. Fracolli, L.A., Reticena, K.D.O., Abreu, F.C.P.D., & Chiesa, A M. (2018). The implementation of a home visits program focused on parenting: an experience report. *Revista da Escola de Enfermagem da USP*, *52*
